# Do males and females respond differently to ocean acidification? An experimental study with the sea urchin *Paracentrotus lividus*

**DOI:** 10.1007/s11356-020-10040-7

**Published:** 2020-07-10

**Authors:** Tihana Marčeta, Valerio Matozzo, Silvia Alban, Denis Badocco, Paolo Pastore, Maria Gabriella Marin

**Affiliations:** 1 Department of Biology, University of Padova, via Ugo Bassi 58/B, 35131 Padova, Italy; 2 Present Address: Institute of Marine Sciences (ISMAR), CNR, Venezia, Italy; 3 Department of Chemical Sciences, University of Padova, Via Marzolo 1, 35131 Padova, Italy

**Keywords:** Sea urchins, Ocean acidification, Sex, Biomarkers, Physiological parameters, Gonadosomatic index, Righting time

## Abstract

Seawater pH lowering, known as ocean acidification, is considered among the major threats to marine environment. In this study, post-spawning adults of the sea urchin *Paracentrotus lividus* were maintained at three pH values (8.0, 7.7, 7.4) for 60 days. Physiological, biochemical, cellular, behavioural and reproductive responses were evaluated in males and females. Significant differences between sexes were observed, with higher ammonia excretion and lower catalase activity in males. Respiration rate (after 21 days), catalase activity in gonads and total coelomocyte count showed the same increasing trend in males and females under low pH. Ammonia excretion, gonadosomatic index and lysozyme activity exhibited opposite responses to low pH, with an increasing trend in males and decreasing in females. Results demonstrated that exposure to low pH could result in different response strategies of male and female sea urchins at a physiological, biochemical and immunological level. Reduced female gonadosomatic index under low pH suggested decreased energy investment in reproduction.

**Electronic supplementary material:**

The online version of this article (10.1007/s11356-020-10040-7) contains supplementary material, which is available to authorized users.

## Introduction

Ocean acidification (OA) is a phenomenon of lowering seawater pH, due to the dissolution of rising atmospheric CO_2_. Alterations of atmospheric gas composition observed during the last two centuries are mainly originated from anthropogenic activities, first from all fossil fuels’ combustion. Since pre-industrial time, ocean surface pH has decreased by approximately 0.1 units (IPCC 2013). The present average pH value for shallow and surface seawaters is 8.1, and predicted global surface pH reduction is of 0.06–0.32 units by the year 2100 and 0.7 units by 2300 (Hartin et al. 2016; IPCC 2019).

Responses to OA represent a species-specific phenomenon, and the effects detected in laboratory experiments are dependent on geographic area and life-history stages of the studied species (Hall-Spencer et al. 2015), as well as on the duration of the experimental exposure (Suckling et al. 2015). There are evidence that OA can differentially affect physiology, reproduction, biochemistry and survival of marine invertebrates (McClellan-Green et al. 2007; Ellis et al. 2014; Lane et al. 2015). When exposed to OA, organisms may change their resource energy allocation and due to higher production cost of eggs compared to sperm, females could be deemed more vulnerable to this stressor. Notwithstanding, Ellis et al. (2017) reported in their review that only 3.77% of 511 OA studies published between January 2008 and May 2016 tested gender-related responses in fish, crustaceans, echinoderms and molluscs. When tested, sex significantly influenced the response to OA, suggesting that sex has to be considered in order to correctly evaluate the impact at the population level (Ellis et al. 2017).

Among aquatic species, echinoderms are considered as model organisms to assess the effects of changing environmental conditions. Echinoderms include exclusively marine species, such as sea urchins, which build calcareous skeleton in both larval and adult phase. Skeletal rods in larvae and test, teeth and spines in adults are formed from magnesium calcite that is one of the most soluble forms of calcium carbonate (Morse et al. 2006). In long-term experimental exposures to OA, sea urchins revealed mostly negative but sub-lethal effects, with diminished calcification as principal impairment in both adult and larval stages. Although sea urchin species inhabiting low pH environments such as upwelling regions, intertidal pools, and CO_2_ vents exhibited great potential to adapt to OA, in about 20 species of echinoplutei from several world regions and habitats, reduction in growth and increased alteration of body morphology were observed (Byrne and Hernández 2020). Long-lasting experiments can allow us to inspect organisms’ capability to acclimate to low pH and to produce more relevant data for predicting long-term consequences of OA. It was demonstrated that short-term exposures to low pH values could lead to hypercapnic conditions in sea urchin coelomic fluid (Miles et al. 2007; Spicer et al. 2011; Dupont and Thorndyke 2012; Spicer and Widdicombe 2012; Stumpp et al. 2012; Holtmann et al. 2013; Kurihara et al. 2013). This effect could represent a “shock” response (Byrne 2012; Queirós et al. 2015) possibly related to both the absence of respiratory pigments and low capability of sea urchins to regulate ions. Generally, in most species, with few exceptions (Kurihara et al. 2013), the acid-base balance is recovered in some days or weeks (Calosi et al. 2013; Dupont and Thorndyke 2012; Stumpp et al. 2012; Holtman et al. 2013; Moulin et al. 2014). Experiments on *Paracentrotus lividus* (from 6 days to 2 months long) showed an increased coelomic fluid buffer capacity under low pH. However, in this species, coelomic fluid pH seems to be only partially compensated at extreme pH condition (7.4 pH). This compensation of the coelomic fluid pH in *P. lividus* was not dependent on skeleton dissolution, indeed skeletal mechanical properties were not affected at 7.7 pH (Catarino et al. 2012; Collard et al. 2013, 2014, 2016; Cohen-Rengifo et al. 2019). The acid-base regulation capability allows sea urchins to maintain appropriate extracellular and intracellular pH conditions, but the processes involved in this regulation are energy-consuming and lead to increased oxygen uptake. Therefore, other processes such as growth, reproduction and behaviour could be compromised if energy acquisition is not increased (Dupont et al. 2013).

It is supposed that increased levels of CO_2_ cause oxidative stress directly by increasing the production of ROS and/or indirectly by lowering internal pH, which may induce the release of chelated transition metals such as Fe^2+^ from intracellular compartments and enhance the Fenton reaction (Tomanek et al. 2011). Induction of oxidative stress by low pH has been scarcely investigated in marine echinoderms (Migliaccio et al. 2019), but it was evaluated and detected in other taxa, such as marine bivalves (Tomanek et al. 2011; Matozzo et al. 2013; Benedetti et al. 2016; Velez et al. 2016; Freitas et al. 2017a; Nardi et al. 2017; Sui et al. 2017; Huang et al. 2018; Munari et al. 2018), crustaceans (Priya et al. 2017; Rato et al. 2017; Glippa et al. 2018), polychaetes (Freitas et al. 2017b), gastropods (Cardoso et al. 2017), corals (Soriano-Santiago et al. 2013) and fish larvae (Pimentel et al. 2015). In all these studies, the exposure to OA conditions lasted from at least 72 h to a maximum of 1 month. For the sea urchin *P. lividus*, seasonal changes in biomarker responses were observed in specimens from the Gulf of Annaba (Algeria), with an increase in antioxidant enzymes’ activity during the reproductive period (spring), suggesting that for this species natural physiological cycle as well as biotic and abiotic factors could influence oxidative stress responses (Amri et al. 2017).

As demonstrated in *Lytechinus variegatus*, *Echinometra lucunter* and *Strongylocentrotus droebachiensis*, ocean acidification affects adult sea urchin immune system after a short-term exposure (from 24 h to 7 days). Alterations of immunological parameters seem to be mainly linked to pH decrease in coelomic liquid and appeared reversible when natural values were re-established (Dupont and Thorndyke 2012; Leite Figueiredo et al. 2016).

Effects of OA on sea urchin fecundity was evaluated mainly by measurement of gonadic mass production (Siikavuopio et al. 2007; Stumpp et al. 2012; Kurihara et al. 2013; Taylor et al. 2014; Uthicke et al. 2014; Mos et al. 2016; Dworjanyn and Byrne 2018), as well as by determination of egg number and size, gamete performance or RNA/DNA ratio in gonads (Catarino et al. 2012; Cohen-Rengifo et al. 2013; Dupont et al. 2013; Uthicke et al. 2013; Suckling et al. 2014; Graham et al. 2015; Campbell et al. 2016; Karelitz et al. 2020).

In the present study, adults of the sea urchin *P. lividus* were used as model organism. This species is widely distributed in the Mediterranean and in the north-eastern Atlantic (Boudouresque and Verlaque 2013), where it plays a dominant role as a grazer and acts as a keystone species in controlling dynamic, structure and composition of infralittoral macroalgal assemblages (Hereu 2006; Privitera et al. 2008; Boudouresque and Verlaque 2013). During a 60-day experiment, post-spawning adults of *P. lividus* were exposed to control pH (8.0) and to two reduced pH values, 7.7 and 7.4, according to projections for shallow and surface seawaters by 2100 and 2300, respectively (Hartin et al. 2016; IPCC 2019). A number of physiological, biochemical, cellular, behavioural and reproductive responses were investigated in both males and females. The hypotheses we tested were as follows: (i) OA affects the biological responses measured in *P. lividus*; (ii) males and females respond differently to OA.

## Materials and methods

### Specimen collection

About 200 adult specimens of *P. lividus*, with a live weight of 36.7 ± 11.4 g and a test diameter of 4.5 ± 0.5 cm, were collected by SCUBA divers at approximately 5 m depth in the southern basin of the Venice Lagoon (NW Adriatic Sea, Italy) between February and March 2017. The sampling area is close to the southern inlet of the lagoon where pollution levels are generally low (Parolini et al. 2010; Parolini et al. 2012; Cassin et al. 2018; Zonta et al. 2018). In the laboratory, animals were acclimated in flow-through aquaria for 2 weeks at least, at 18 ± 0.5 °C temperature and 34 ± 1 salinity, and fed with *Ulva* sp. In order to recognize male and female sea urchins and to obtain individuals in the same very early stage of gametogenesis, all specimens were induced to spawn, by injecting 0.5 ml of 0.5 M KCl solution into the coelom, through the peristome membrane (Gago and Luís 2011). After that, males and females were maintained in separate aquaria and allowed to recover for a week in the previously reported conditions. Prior to exposure, the sea urchins were acclimatized to the experimental conditions by gradually reducing the natural pH values by about − 0.3 or − 0.6 units (approximately 0.1 reduction per day).

### Experimental sea urchin culture system

Post-spawning adults of *P. lividus* were kept under three pH values: 8.0, 7.7 and 7.4. Each experimental condition was assessed in triplicate 60 l tanks, each containing at least six males and nine females separated by a plastic grid. Tanks were supplied with filtered seawater (5 μm) at a flow rate of 300 ml min^−1^ and were equipped with an aerator. In each individual low pH (7.7 and 7.4) tank, the pH value was maintained by bubbling CO_2_ using an electronic control system (Aquarium Controller Evolution, mod. ACQ110, Aquatronica, Italy) connected to a pH electrode (ACQ310N-pH by Aquatronica, Italy). To verify and adjust pH electrode measurements, in each experimental tank pH value was checked twice a day at least using a benchtop pH-meter Basic 20+ (Crison, Spain) calibrated daily with Crison buffer solutions.

In order to promote the gonadal maturation, sea urchins were fed ad libitum with fresh *Ulva* sp., and maintained at 18 °C temperature (Grosjean et al. 1998), and 9 h light: 15 h dark photoperiod (Spirlet et al. 2000). Salinity values were in the same range measured during the acclimation period. Throughout the experiment, no spontaneous spawning event was observed.

At each experimental condition, sea urchins were randomly sampled within the tanks and physiological effects were evaluated after 7, 14, 21 and 40 days through measurements of respiration rate, ammonia production rate and assimilation rate. Due to some technical inconveniences, data were not collected at day 40 for ammonia production and at days 21 and 40 for assimilation. For each parameter measured, four males and four females per experimental condition were used. After 40 days, righting time (see below for details) was also measured on six males and six females per experimental condition. Physiological and behavioural measurements were performed at the same pH, temperature and salinity used during exposure. After 60 days of exposure to differing pH values, six males and six females were used to evaluate gonadosomatic index, superoxide dismutase (SOD) and catalase (CAT) activity in gonads and digestive tract, coelomocyte number and volume (TCC and CV respectively) and lysozyme activity in coelomic fluid and coelomocytes.

### Seawater chemistry

On days 30, 50 and 60, seawater samples from each experimental condition were collected into 250 ml polypropylene bottles. In order to halt the biological activity, samples were immediately poisoned with 100 μl of saturated mercuric chloride solution (HgCl_2_) and were stored at 4 °C in the dark until analysis.

Total alkalinity (TA) was determined via potentiometric titration using an automatic titrator (836 Titrando, Metrohom). Each seawater sample was thermostated at 25 °C (HAAKE C25P Phoenix II, ENCO) before titration. A synthetic alkalinity standard was prepared following the method of Dickson et al. (2003) and used as a reference. The estimate of the extended uncertainty interval was 4.9% with respect to the TA value of the standard. All TA values obtained with the synthetic alkalinity standard in the analysis of the real samples were compliant. The 4.3% of the titrations of the seawater samples were repeated more than two times to meet the relative precision specifications of 2% obtained in the validation of the TA standard.

The dissolved inorganic carbon (DIC) content, carbonate concentration values and CO_2_ partial pressure (pCO_2_) in seawater were computed at the sampling temperature, salinity and pH_T_ using the TA values. All thermodynamic equilibrium constants were computed according to Millero (1979), Millero (1995) and Millero et al. (2006). The solubility values of calcite and aragonite were obtained from Mucci (1983) and from Ingle (1975). The saturation states of calcite (Ω_ca_) and aragonite (Ω_ar_) were computed based on the solubility products reported in Millero (1979). Results are reported in Table 1.

**Table 1:** Seawater carbonate chemistry variables (mean values ± SD; *n* = 9) recorded during the experiment

Tanks	pH_T_	DIC (μmol kg^−1^)	TA (μmol kg^−1^)	pCO_2_ (μ Atm)	Ω_Ca_	Ω_Ar_
8.0 pH	8.02 ± 0.03	2564.97 ± 110.81	2772.47 ± 120.47	544.02 ± 59.42	5.65 ± 0.12	3.68 ± 0.09
7.7 pH	7.69 ± 0.02	2703.25 ± 25.32	2780.86 ± 27.43	1263.29 ± 41.99	2.95 ± 0.17	1.92 ± 0.11
7.4 pH	7.38 ± 0.02	2783 ± 43.71	2757.13 ± 42.10	3643.17 ± 88.35	1.47 ± 0.05	0.95 ± 0.03

*TA*, total alkalinity; *DIC*, total dissolved inorganic carbon; *pCO*_*2*_, CO_2_ partial pressure; *Ωca*, calcite saturation state; *Ωar*, aragonite saturation state

### Respiration rate

Since feeding increases the buffer capacity and non-fed individuals can undergo severe metabolic acidosis (Stumpp et al. 2012; Collard et al. 2013), sea urchins were not fasted before respiration measurements to reproduce the environmental conditions more faithfully and to avoid additional stress. To measure oxygen consumption, individual sea urchins were placed in 0.8 l plexiglas respirometry chambers provided with a magnetic stirrer bar placed under a perforated plate. Chambers were filled with air-saturated 0.45 μm-filtered seawater at each pH studied and placed on the multi-position magnetic stirrer. During measurements, the chambers were kept at 18 °C using a thermostatic bath. For each batch, a chamber without sea urchin was used as a control. Oxygen concentration was measured after 0, 30, 60 and 90 min using an optical oxygen meter (fibre-optic oxygen meter‑Piccolo2, Pyro Science GmbH, Aachen, Germany). Oxygen saturation never fell below 70% during the trial. Oxygen uptake (μmolO_2_ h^−1^ g^−1^) was calculated by multiplying the slope of the oxygen depletion curve by the volume of seawater inside the chamber and dividing by the live sea urchin weight. The volume of water was determined by subtracting the volume of each sea urchin from the total volume in the chamber.

### Ammonia excretion

Ammonia excretion (μmolN-NH_3_ h^−1^ g^−1^) was measured in water samples collected from each respirometry chamber after 90 min. Ammonia was determined spectrophotometrically according to the method of Solorzano (1969). Ammonia excretion was calculated from the difference in ammonia concentration between the chambers with and without animals and referred to sea urchin live weight.

### Assimilation efficiency

To measure assimilation efficiency, sea urchins were placed individually in 2.5-l beakers filled with filtered seawater (0.45 μm) for 24 h and during this period they were not fed. Faeces from each beaker were drawn off, filtered on pre-ashed and weighed glass fibre filters (Whatman GFC), and rinsed with distilled water to remove the salt. Filters were then dried in an oven at 60 °C, weighed after 24 h, ashed in a muffle furnace at 450 °C for 4 h and re-weighed (Conover 1966; Reid et al. 2010). Weight determinations were performed using a Mettler Toledo, XS105 Dual Range analytical balance (0.01 mg readability). The same procedure was applied in triplicate on diet samples. In faeces and algae, organic content (OC) was calculated as ash-free dry weight obtained as the difference between dry and ash weight. Absorption efficiency (AE) was determined according to Conover (1966): AE = [(DietOC − FaecesOC) / (1 − FaecesOC) × DietOC] × 100, where DietOC and FaecesOC are the organic fractions in algae and in faeces, respectively.

### Superoxide dismutase and catalase activity

Gonads and digestive tract were dissected from six males and six females and aliquots of the individual tissues were placed in tubes and immediately frozen in liquid nitrogen and stored at − 80 °C until analysis. The gonads and digestive tract were thawed on ice and homogenized (1:4, w:v) in 0.1 M Tris–HCl buffer (pH 7.5) containing 0.15 M KCl, 0.5 M sucrose, 1 mM EDTA and 1 mM dithiothreitol (DTT, Sigma). Homogenates were centrifuged at 12,000*g* for 45 min at 4 °C and supernatants (SN) were collected to measure antioxidant enzyme activities. To this aim, widely validated methods in sea urchins were used in this study (Zuo et al. 2018; Klein et al. 2019; Zapata-Vivenes and Aparicio 2019).

Total SOD activity was measured in SN of gonads and digestive tract with the xanthine oxidase/cytochrome C method in accordance with Crapo et al. (1978). The cytochrome C reduction by superoxide anion generated by xanthine oxidase/hypoxanthine reaction was detected using a Beckman Coulter (DU® Series 730) spectrophotometer at 550 nm at room temperature (20 °C). Enzyme activity was expressed as U mg^−1^ of protein, 1 unit of SOD is defined as the amount of sample producing 50% inhibition of cytochrome C reduction in the assay conditions. The reaction mixture contained 46.5 μM KH_2_PO_4_/K_2_HPO_4_ (pH 8.6), 0.1 mM EDTA, 195 μM hypoxanthine, 16 μM cytochrome c and 2.5 μU xanthine oxidase.

CAT activity was measured in tissue SN following the method described in Aebi (1984). Decreases in absorbance of a 50-mM H_2_O_2_ solution (Ɛ = − 0.0436 mM^−1^ cm^−1^) in 50 mM phosphate buffer (pH 7.8) and 10 μl of tissue SN were continuously recorded at 240 nm and at 10-s intervals for 1 min. The results were expressed as U mg^−1^ of protein, 1 unit of CAT being defined as the amount of enzyme that catalyses the dismutation of 1 μmol of H_2_O_2_ min^−1^.

For both SOD and CAT assays, SN protein concentrations were quantified in accordance with Bradford (1976).

### Coelomic fluid collection

Two millitres of coelomic fluid were collected from the peristomial membrane of each animal, with a plastic syringe, and stored in ice. Coelomic fluid (1 ml) was used to determine the total coelomocyte count (TCC) and coelomocyte volume (CV), while 1 ml was used to measure both lysozyme activity and total protein concentration in coelomocyte lysate (CL) and cell-free coelomic fluid (CFC).

### Total coelomocyte count and coelomocyte volume

A Coulter counter (Z2 mod., Beckman Coulter) was used to determine TCC and CV after adding 1 ml of coelomic fluid to 19 ml of 0.45 μm-filtered seawater. TCC results were expressed as the number of coelomocytes (× 10^6^) ml coelomic fluid^−1^. The haemocyte volume was expressed in femtolitres (fl).

### Lysozyme activity

Lysozyme activity was quantified in both CFC and CL, according to Santarém et al. (1994) and Fernández-Boo et al. (2018). Coelomic fluid from each sea urchin was centrifuged at 780*g* for 10 min. The supernatant, corresponding to CFC, was collected, whereas the coelomocytes were resuspended in distilled water and sonicated at 4 °C for 1 min to obtain CL. CL and CFC were frozen and stored at − 80 °C before analyses. Fifty microlitres of CL and CFC were added to 950 μl of a 0.15% suspension of *Micrococcus lysodeikticus* (Sigma) in 66 mM phosphate buffer, pH 6.2; and the decrease in absorbance (ΔA min^−1^) was continuously recorded at 450 nm for 5 min at room temperature. Standard solutions containing 1, 2.5, 5 and 10 μg lysozyme per ml of 66 mM phosphate buffer, pH 6.2, were prepared from crystalline hen egg-white lysozyme (Sigma). The average decrease in absorbance per minute was determined for each enzyme solution, and a standard curve of enzyme concentration versus ΔA min^−1^ was drawn. Results were expressed as μg lysozyme mg protein^−1^. CL and CFC protein concentrations were also quantified according to Bradford (1976).

### Righting time

Each animal was tested three times in water from its exposure tank. At the beginning of the trial, the sea urchin was placed on its aboral surface and the time used by each animal to right itself completely was recorded. The test was carried out using a 5 l plastic rectangular container with a smooth surface and large enough to avoid contact between vertical walls and the animal. After the first experimental trial, water was completely removed from the container in order to detach the sea urchin without disturbing it. Each individual was maintained in its experimental tank for 1 h before repeating the measurement.

### Gonadosomatic index

Live sea urchins were weighed using a digital balance (± 0.01 g) and dissected to obtain the gonads. Dissected gonads were weighed and GSI was calculated as the percentage of fresh weight of gonads respect to the live weight of the animal.

### Statistical analysis

For all the parameters considered, significant effects due to pH, sex and pH*sex interaction were assessed by linear mixed models with a tank as a random effect and followed by the Tukey post-hoc correction. The threshold for significance was set at *p* < 0.05.

Lastly, a canonical correlation analysis (CCA) was performed using a data matrix made up on all biomarkers’ measurements (i.e., respiration rate, SOD and CAT activity in gonads and digestive tract, total coelomocyte number and volume, lysozyme activity in CL and CFC, righting time and gonadosomatic index) detected in males and females after at least 40 days of exposure. The set of variables included are pH and gender vs the measured physiological, cellular and biochemical parameters. All statistical analyses were performed using package R (R Core Team 2019, Austria) with the CCA package (González and Déjean 2012) and r41sqrt10 package (Finos 2020).

## Results

Detailed statistical results for all biomarkers measured are shown in the Supplementary material.

### Physiological parameters

**Fig. 1:**
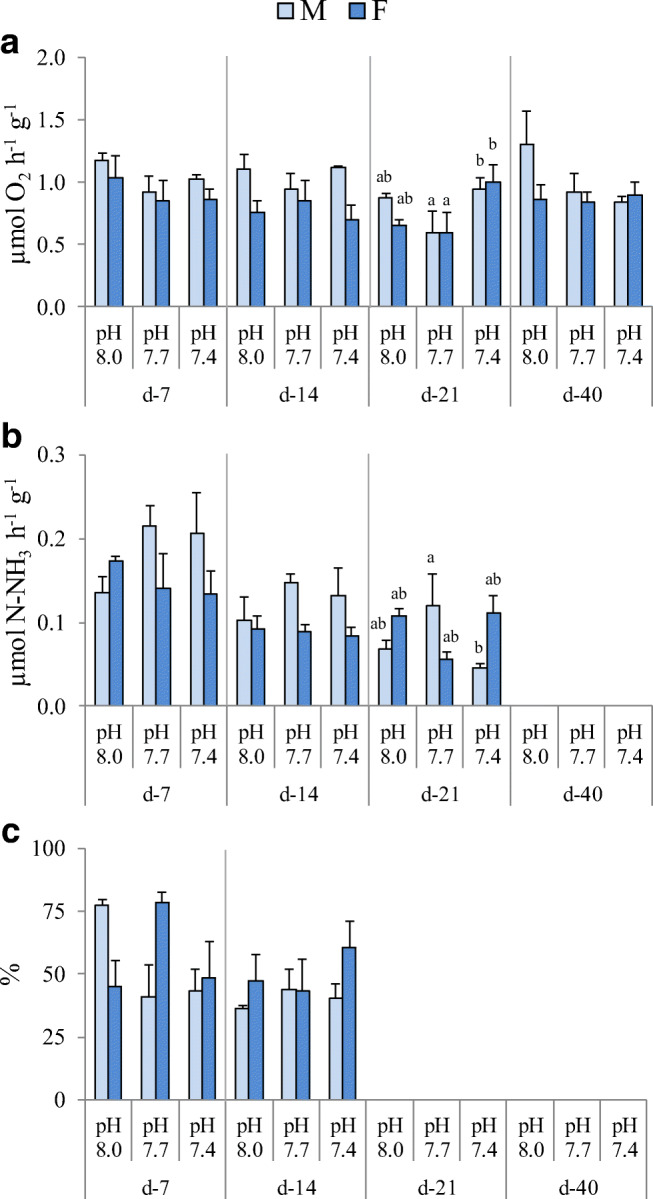
Respiration rate (**a**), ammonia excretion (**b**) and assimilation efficiency (**c**) of *P. lividus* males (M) and females (F) after 7, 14, 21 and 40-day exposure to 8.0, 7.7 and 7.4 pH. Values are the means ± SD (*n* = 3). Significant differences among the various experimental conditions are presented with lower case letters (**a**, **b**). Due to some technical inconveniences, data are not available at day 40 for ammonia production and at days 21 and 40 for assimilation

No effects of the experimental conditions tested were recorded in respiration rate after 7 and 40 days of exposure (Fig. 1a, Table 2). After 14 days, this parameter was significantly affected by gender and showed higher values in males compared with females. A significant effect of pH was recorded after 21 days of exposure, with a significant increase in respiration rate at 7.4 pH respect to 7.7 pH in both males and females.

**Table 2:** Linear mixed model results for respiration rate, ammonia production and assimilation efficiency in *P. lividus*, after 7, 14, 21 and 40 days of exposure to 8.0, 7.7 and 7.4 pH. Due to some technical inconveniences, data are not available at day 40 for ammonia production and at days 21 and 40 for assimilation. Significant effects are in italics

	Factor		d-7	d-14	d-21	d-40
Respiration rate	pH	*p*	0.341	0.892	*0.000*	0.182
	sex	*p*	0.336	*0.005*	0.386	0.214
	pH*sex	*p*	0.914	0.256	0.350	0.268
Ammonia production	pH	*p*	0.855	0.806	0.799	
	sex	*p*	0.353	*0.043*	0.354	
	pH*sex	*p*	0.303	0.677	*0.001*	
Assimilation efficiency	pH	*p*	0.322	0.660		
	sex	*p*	0.689	0.220		
	pH*sex	*p*	*0.008*	0.593		

No effects of the variables considered on ammonia excretion were observed at 7 days (Table 2). As in respiration rate, a significant effect of sex was observed after 14 days, with higher values in males than in females. After 21 days, ammonia excretion pH*sex interaction was significant. In details, a marked decrease in ammonia excretion was recorded in males kept at 7.4 pH, whereas the same was observed in females maintained at 7.7 pH (Fig. 1b). A significant effect of interaction between pH and sex on assimilation efficiency was detected after 7 days of exposure (Table 2). Under low pH values, this parameter showed decreased values in males, while an increase was observed in females, mostly at 7.7 pH (Fig. 1c).

### Antioxidant enzyme activities

**Fig. 2:**
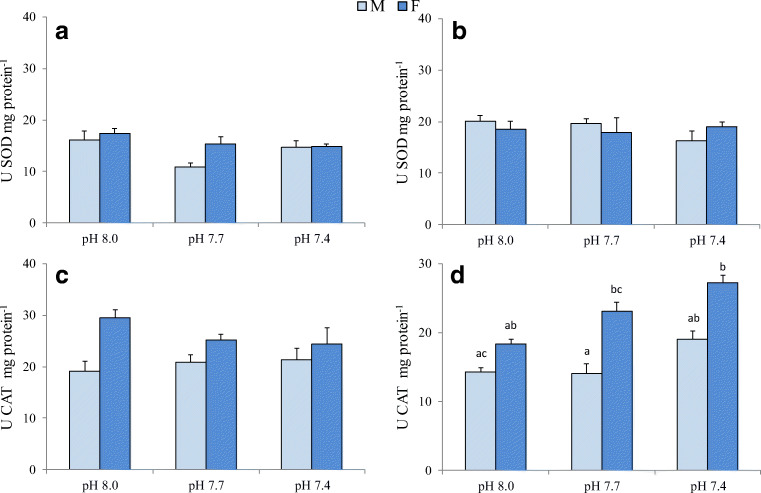
SOD and CAT activity in gonads (**a**, **c**) and digestive tract (**b**, **d**) of *P. lividus* males (M) and females (F) after 60-day exposure at 8.0, 7.7 and 7.4 pH. Values are the means ± SD (*n* = 3). Significant differences among the various experimental conditions are presented with lower case letters (**a**, **b**)

In both gonads and digestive tract, pH, sex and their interaction did not affect significantly SOD activity (Fig. 2a, b, Table 3). Conversely, CAT activity in gonads was significantly affected by sex (Table 3), showing higher values in females than in males (Fig. 2c). In the digestive tract, both pH and sex influenced significantly CAT activity (Table 3). In these tissues, an increasing trend in CAT activity was observed at low pH with higher values in females. In particular, the activity was significantly higher in females at 7.4 pH with respect to males at 8.0 and 7.7 pH (Fig. 2d).

**Table 3:** Linear mixed model results for superoxide dismutase (SOD) and catalase (CAT) activities in gonads and digestive tract, total coelomocyte count (TCC) and coelomocyte volume (CV), lysozyme activity (Lyso) in cell-free coelomic fluid (CFC) and coelomocytes (CL), righting time (RT) and gonadosomatic index (GSI) in *P. lividus*. Significant effects are in italics

		SOD		CAT				Lyso			
Factor		Gonad	Dig. tract	Gonad	Dig. tract	TCC	CV	CFC	CL	RT	GSI
pH	*p*	0.159	0.637	0.887	0.011	*0.011*	0.801	0.110	0.511	0.256	0.429
sex	*p*	0.198	0.902	*0.017*	*0.00*	0.381	0.465	0.459	0.884	0.375	0.232
pH*sex	*p*	0.534	0.363	0.440	0.506	0.239	0.588	*0.002*	*0.034*	0.361	*0.002*

### Coelomocyte and coelomic fluid parameters

**Fig. 3:**
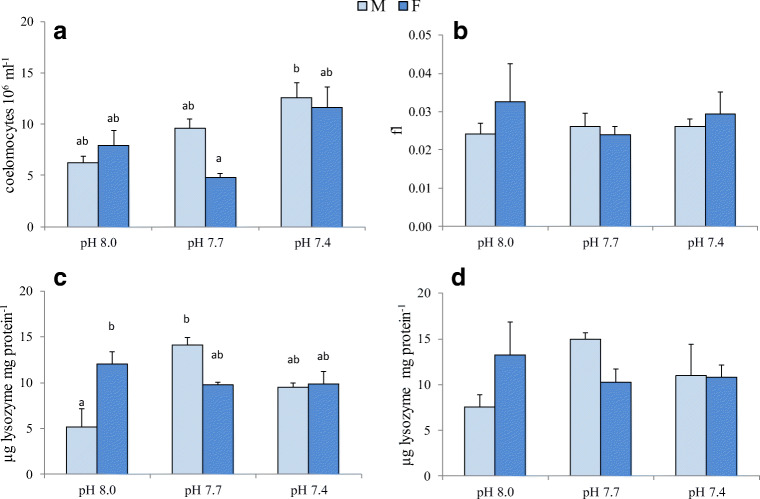
Total coelomocyte count, TCC (**a**), coelomocyte volume, CV (**b**), lysozyme activity in CFC (**c**) and in CL (**d**) in *P. lividus* males (M) and females (F) after 60-day exposure to 8.0, 7.7 and 7.4 pH. Values are the means ± SD (*n* = 3). Significant differences among the various experimental conditions are presented with lower case letters (**a**, **b**)

TCC was significantly affected by pH (Table 3). Higher TCC values were observed at 7.4 pH in both sexes, in particular males at 7.4 pH showed significantly higher coelomocyte number compared to females at 7.7 pH (Fig. 3a). On the contrary, CV values were not significantly influenced by the experimental conditions tested (Fig. 3b, Table 3). In both CFC and CL, lysozyme activity was significantly affected by pH*sex interaction (Table 3). Interestingly, lysozyme activity showed the same pattern of variation in CFC and CL (Fig. 3c, d). At 8.0 pH, females showed significantly higher CFC lysozyme activity compared to males. No pH-induced variations in CFC lysozyme activity were observed in females, whereas in males a significant increase of the enzyme activity was observed at 7.7 pH respect to control (Fig. 3c).

### Righting time and gonadosomatic index

**Fig. 4:**
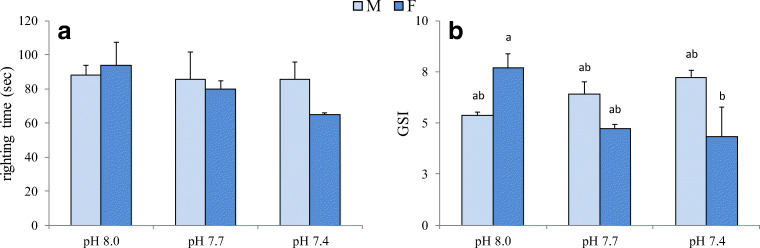
Righting time (**a**) and gonadosomatic index (**b**) of *P. lividus* males (M) and females (F) after 60-day exposure to 8.0, 7.7 and 7.4 pH. Values are the means ± SD (*n* = 3). Significant differences among the various experimental conditions are presented with lower case letters (**a**, **b**)

Although no significant effects of the experimental conditions were found (Table 3), a slight decrease in righting time was shown in females under reduced pH, but not in males (Fig. 4a). GSI values were significantly influenced by pH*sex interaction (Table 3). At decreasing pH, GSI showed an opposite trend in the two sexes, slightly increasing in males and markedly decreasing in females (Fig. 4b). Females maintained at 7.4 pH showed a significantly lower GSI compared with females from the control condition.

### Canonical correlation analysis

**Fig. 5:**
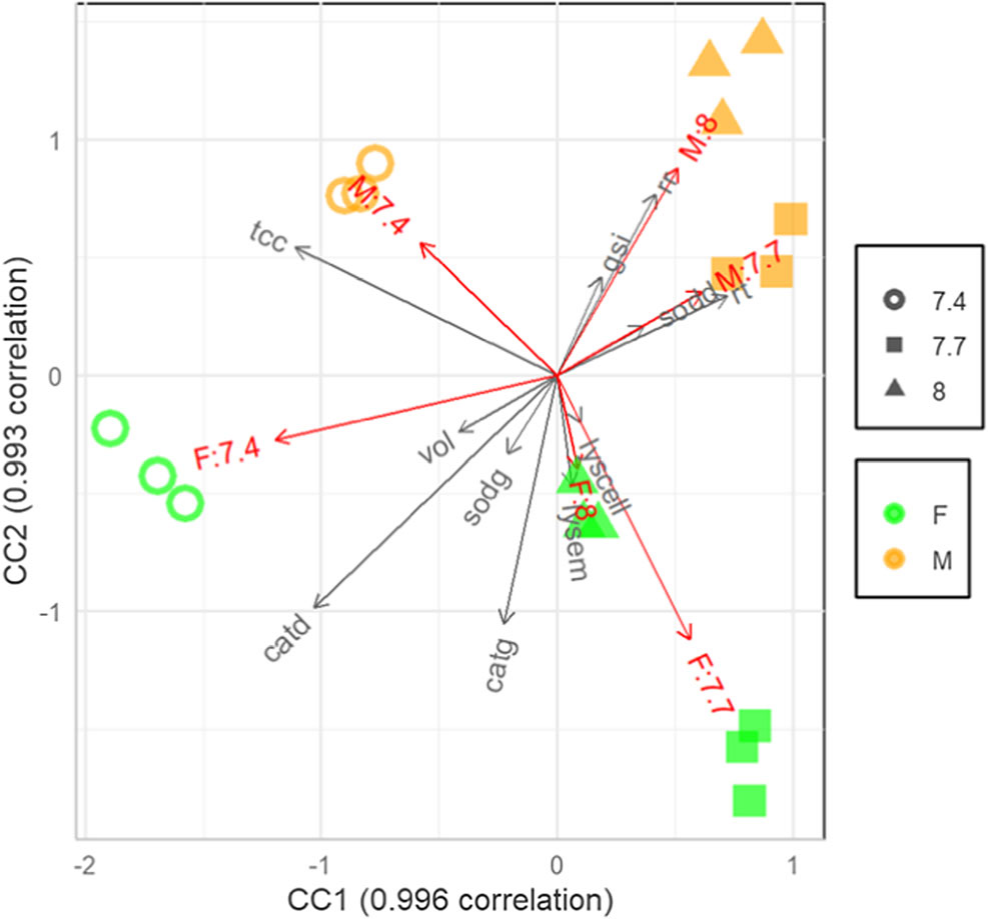
Canonical correlation analysis of the biomarker dataset. M:8.0, M:7.7, M:7.4: males at pH 8.0, 7.7 and 7.4, respectively; F:8.0, F:7.7, F:7.4: females at pH 8.0, 7.7 and 7.4, respectively. Abbreviations: respiration rate (rr), superoxide dismutase (sod), catalase (cat), gonads (g), digestive tract (d), total coelomocyte count (tcc), coelomocyte volume (vol), lysozyme (lys), cell-free coelomic fluid (em), coelomocytes (cell), righting time (rt) and gonadosomatic index (gsi)

The CCA performed on the whole dataset of biomarkers measured from day 40 to day 60 is shown in Fig. 5. The explained correlation is 99% for both canonical correlations. The CCA biplot reveals two clear pairs of canonical components. The first canonical component is roughly associated with the pH level, while the second is associated with the gender. There is a clear separation of pH 7.4 in both males and females. In particular, control females and those at 7.7 pH are well discriminated, while there is a less evident separation between the same pH conditions in males.

## Discussion

The increased CO_2_ concentration in seawater can affect marine organisms both directly, as CO_2_ enters the organisms by diffusion inducing hypercapnia (i.e., CO_2_ accumulation in the internal fluids) and indirectly through acidosis (i.e., internal pH decrease). In sea urchins, regulation of internal pH under seawater acidification is documented in both laboratory and natural conditions (Catarino et al. 2012; Dupont and Thorndyke 2012; Collard et al. 2013; Leite Figueiredo et al. 2016; Lewis et al. 2016; Migliaccio et al. 2019). Maintenance of acid-base homeostasis is attained by increasing bicarbonate levels in the coelomic fluid, as demonstrated in *P. lividus* and *S. droebachiensis* exposed to reduced pH (Collard et al. 2013; Stumpp et al. 2012). However, this is an energy-requiring process possibly leading to a reduction in the energy that can be allocated to other processes, such as growth and reproduction (Melzner et al. 2009; Collard et al. 2013). In this context, variations in physiological responses as a proxy for metabolic expenditure have been assessed in our study. After 21 days of exposure to reduced pH, *P. lividus* respiration rate decreased at 7.7 and increased at 7.4 pH, the difference resulting significant between the two reduced pH conditions in both males and females. In the same species, increased respiration rate was observed by Catarino et al. (2012) after 19 days of exposure to reduced pH (7.7 and 7.4) at a temperature of 10 °C, whereas no differences respect to controls were found at 16 °C. Although in our study pH significantly influenced respiration rate after 21 days, no significant effects of pH were observed after 40 days of exposure. This suggested that both male and female *P. lividus* have the potential to acclimate at low pH conditions after prolonged exposure, as already observed for the same species, both in a laboratory 2-months exposure (Cohen-Rengifo et al. 2019) and in specimens from CO_2_ vents at Ischia Island (Migliaccio et al. 2019). Similarly, in other species, namely *E. mathaei*, *Strongylocentrotus fragilis*, *Strongylocentrotus droebachiensis*, and *Echinometra* sp. A, respiration rate was not affected by reduced pH following long-term exposures (from 49 to 140 days) (Stumpp et al. 2012; Moulin et al. 2014; Taylor et al. 2014; Uthicke et al. 2014). In addition, results obtained after prolonged exposure (more than 5 months) highlighted the capability of *Hemicentrotus pulcherrimus* and *Sterechinus neumayeri* to acclimate to low pH conditions (Kurihara et al. 2013; Suckling et al. 2015). However, significant effects of low pH on sea urchin metabolic rate are reported in the literature, following exposures that lasted no longer than 5 months. For example, in *Echinometra mathaei* and *Anthocidaris crassispina*, oxygen uptake was found significantly reduced under OA conditions lasted 42 and 140 days, respectively (Uthicke et al. 2013; Wang et al. 2013). Conversely, in *Sterechinus neumayeri* and *Heliocidaris erythrogramma* oxygen consumption increased after exposure for 30, and 60 days to reduced pH (Suckling et al. 2015; Carey et al. 2016). Overall, these data highlight that capability of sea urchins to acclimate to low pH is species- and exposure duration-dependent.

Ammonia production is an indicator of protein metabolism and it was hypothesized that under acidified conditions increased ammonia excretion could act as an additional acid extrusion mechanism in mussels (Thomsen and Melzner 2010) and sea urchins (Stumpp et al. 2012). In this study, after 14 days of exposure, ammonia production was not significantly affected by pH, but it was significantly different between sexes with higher values in males. However, after 21 days, the effect of pH was significant, with decreasing trend at low pH conditions in both sexes, even though ammonia excretion decreased at 7.4 pH in males and at 7.7 pH in females. Unfortunately, ammonia excretion data from 40-day-exposed sea urchins lack in our study. Consequently, a more exhaustive conclusion about such physiological parameter cannot be formulated. In this regard, it is important to highlight that exposure for 70 days to 860–940 μAtm CO_2_ did not affect ammonia production in *Echinometra* sp. A (Uthicke et al. 2014). Similar results were obtained in *P. lividus* from the vent and non-vent areas at Ischia Island (Migliaccio et al. 2019).

In our study, assimilation efficiency was the only physiological parameter significantly affected after 7 days of exposure. Indeed, a significant interaction between pH and sex was found, suggesting higher assimilation of organic matter from the food by females when exposed to low pH, while an opposite tendency was observed in males. Similarly to what was observed in males in this study, Siikavuopio et al. (2007) reported decreased assimilation in *S. droebachiensis* exposed for 56 days to 6.98 pH. Conversely, in the same species Stumpp et al. (2012) found that the assimilation efficiency was not affected after 10- and 45-days exposure to moderate (1007–1431 μAtm) and high (2800–3800 μAtm) pCO_2_.

Maintenance of antioxidant and immune defence is essential to ensure animal health in acidified seawater. SOD and CAT are considered the primary antioxidant enzymes. They prevent oxidative damage removing reactive oxygen species (ROS) produced during normal metabolism and after oxidative injury. In particular, SOD dismutates superoxide anion (O_2_^−^) to hydrogen peroxide (H_2_O_2_) and O_2_, and CAT is the most important H_2_O_2_ scavenger in cells and reduces H_2_O_2_ to water and O_2_. In this study, SOD activity in both gonads and digestive tract did not vary significantly owing to either pH exposure or animal sex. In the study of Amri et al. (2017), a seasonal assessment of antioxidant activities in gonads of *P. lividus* showed highest levels of SOD activity in spring, when GSI reached its maximum value. Conversely, our results did not highlight a similar relationship between SOD activity and GSI. Unlike SOD, CAT activity was significantly different between sexes, with higher values in both gonads and digestive tract from females. Moreover, gonad CAT activity and GSI exhibited a similar pattern of variation in both females and males, suggesting increasing enzyme activity with increasing gonad development. The important role of CAT in gonad antioxidant defence is mirrored in higher activity levels respect to SOD, as observed in this and in previous studies on *P. lividus* (Perez-Trigo et al. 1995). A prevailing role of CAT against oxidative stress is also confirmed in digestive tract results. Indeed, in both sexes, the enzyme activity increases with increasing stress conditions due to low pH. Contrary to what was observed by Amri et al. 2017, in this study increased CAT activity does not match increased SOD activity. Interestingly, under low pH and high-temperature values, a general increase in CAT activity was also shown in two bivalves, the clam *Chamelea gallina* and the mussel *Mytilus galloprovincialis* (Matozzo et al. 2013). To support the statement of responsiveness of CAT activity to environmental stressors, it is important to highlight that increased enzyme activity was found in gonads of *P. lividus* from areas subject to several industrial activities, and the increase was higher in male specimens (Boussoufa et al. 2017). Although sex-related differences in oxidative stress responses to increased temperature and reduced pH have recently been reported for the marine gastropod *Trochus histrio* (Grilo et al. 2018), similar information is lacking for sea urchins, to our knowledge at least.

The sea urchin coelomic fluid contains coelomocytes, circulating cells that have various roles, ranging from immunity to metabolite transport (Endean 1966). Coelomocytes are involved in the immune defence through several processes, such as phagocytosis, coagulation, encapsulation, cytotoxicity and production of antimicrobial agents and other humoral factors (Silva 2013). Coelomocyte number and cell type proportion, as well as many functional responses, vary with the species and the physiological conditions of individuals, as a response to environmental factors, pollutants, pathogens or accidental injuries (Matranga et al. 2000; Pinsino et al. 2007; Ramírez-Gomez et al. 2010). Exposure of *P. lividus* to pollutants, such as lindane and zinc, or accidental injuries induced an increase in red spherula cells, considered as primary cells possibly affected by stressful conditions (Matranga et al. 2000; Pagliara and Stabili 2012; Stabili and Pagliara 2015).

Among environmental stressors, the effects of exposure to near-future OA on the sea urchins’ immune parameters have been investigated in some recent studies. Alterations in coelomocyte proportions/number and immune functions, such as phagocytic capacity, cell spreading, bacterial growth inhibition capacity, total antioxidant capacity, and nitric oxide production, have been reported (Dupont and Thorndyke 2012; Brothers et al. 2016; Leite Figueiredo et al. 2016; Migliaccio et al. 2019). In this study, no differences in coelomocyte number and volume between sexes were found, but 60-day exposure induced a significant increase in coelomocyte number of both males and females in the extreme experimental condition tested (7.4 pH). Similarly, a significant increase in coelomocyte number with no differences in cell-type proportions was observed in *L. variegatus* exposed for 5 days to 7.3 pH (Leite Figueiredo et al. 2016). Despite coelomocyte number remained unchanged in *Echinometra lucunter* and *E. droebachiensis*, exposure to low pH induced an increase in phagocytic amoebocytes and a decrease in vibratile cells (Dupont and Thorndyke 2012; Leite Figueiredo et al. 2016). No differences in coelomocyte number and type proportion were found in *P. lividus* from CO_2_ vents and control sites at Ischia Island, even though enhanced defensive abilities were revealed in specimens living under reduced pH (Migliaccio et al. 2019). Among immunomarkers, lysozyme, one of the most important lysosomal hydrolase, is synthesized in coelomocytes and released into the coelomic fluid as a defence mechanism against pathogens and other foreign substances (Stabili et al. 1996). In this study, in both cell-free coelomic fluid and coelomocytes, a significant interaction between pH and sex induced an opposite pattern of variation in males and females when exposed to low pH, with increased activity in males, at 7.7 pH in particular, and decreased activity in females. In control conditions, higher lysozyme activity in females respect to males suggested a better immunosurveillance possibly related to different reproductive requirements in the two sexes. Our results match those of Arizza et al. (2013) reporting higher levels of immune activities (cytotoxic, haemolytic and agglutinating) in females of *P. lividus*, together with a higher number of coelomocytes. Since in our study TCC did not differ significantly in females and males, higher lysozyme activity in females seems to be constitutive. Under reduced pH, increased levels in male CFC lysozyme activity in the absence of pathogen challenge could be due to reduced membrane stability of coelomocytes, even though attempts to increase immunosurveillance at the peripheral level cannot be excluded. Unlike males, females exposed to low pH showed decreased lysozyme activity levels in both CL and CFC, suggesting a reduction in energy expenditure through decreased lysozyme secretion. Different strategies in males and females coping with ocean acidification likely occur because of different constitutive levels of the enzyme in the two sexes.

The righting response reflects the general physiological state of the echinoderms when subject to environmental changes and it has been used as an indicator of stress and organism well-being (Lawrence and Cowell 1996). This behaviour is neuromuscular-mediated and represents the coordination ability between tube foot and spine (Bayed et al. 2005). In *P. lividus*, righting time was shown to be possibly related to the reproductive status, with increasing values during gonad development and decreasing values after spawning (Bayed et al. 2005). Other authors highlighted the presence of sublethal effects on *P. lividus* righting time, due to oil pollution (Axiak and Saliba 1981). In our experiment, at the control condition, both male and female righting time was close to the reference value (100 s) reported by Axiak and Saliba (1981) for *P. lividus*. At low pH values, in males righting time remained unchanged, while in females it was reduced on average to 75 s at 7.7 pH, and 69 s at 7.4 pH. However, the pH effect observed in females was not statistically significant suggesting that reduced pH does not affect righting time in *P. lividus*, as observed in juveniles and adults of *L. variegatus* (Challener and McClintock 2013; Emerson et al. 2017). Only extremely low pH (6.6) negatively affected righting time in *S. fragilis* (Taylor et al. 2014). Other behavioural aspects in *P.lividus* were recently investigated by Cohen-Rengifo et al. (2019). They showed that podia adhesion strength was not influenced by reduced pH and highlighted positive synergistic effects of ocean acidification and ocean warming on sea urchin moving velocity at 7.7 pH, but not at 7.4 pH. It was hypothesized that the observed behavioural modifications were ascribed to the accumulation of HCO_3_^−^ in extracellular fluid, as protection against acidosis, and to the compensatory reduction of Cl^−^ (Stumpp et al. 2012; Collard et al. 2014). Alterations in the concentration of these ions could influence GABA receptors, which are involved in neurological mechanisms, such as information processing (Cohen-Rengifo et al. 2019). Potential modifications of sea urchin behaviour under reduced pH deserve further attention in future studies. Interestingly, in our study female righting time and GSI exhibited the same pattern of variation with decreasing pH value.

According to Luís et al. (2005), 2 months were enough to allow gonad maturation and spawning in *P. lividus* after a previous KCl spawning induction, and the observed GSI values were in the range reported for ripe sea urchins in natural populations (Gago et al. 2003). In sea urchins, gonad development is affected by various abiotic and biotic factors, such as temperature (Delorme and Sewell 2016; Zhao et al. 2016; Yeruham et al. 2019), pH (Stumpp et al. 2012; Kurihara et al. 2013; Taylor et al. 2014), photoperiod (Shpigel et al. 2004), food availability and quality (Murillo-Navarro and Jiménez-Guirado 2012; Prato et al. 2018), hydrodynamism (Gianguzza et al. 2013) and pollutants (Schäfer and Köhler 2009; Rouane-Hacene et al. 2018). Sea urchin gonads are very plastic organs that can be used as energy storage: they can be filled or depleted depending on animal conditions. Under stressful conditions, the effects observed are often species-specific and mostly dependent on the type and duration of the stress occurred. Although no differences in GSI are generally reported in males and females from natural populations (Guettaf and San Martin 1995; Gago et al. 2003; Luís et al. 2005), there is increasing evidence of sex-related differences under stressful conditions. Accordingly, in this study, no difference between sexes was found at 8.0 pH; however, an opposite effect on GSI was observed in the two sexes at low pH, with a significant decrease at 7.4 pH in females and an increasing trend at low pH values in males. In a previous study, RNA/DNA ratio, an indicator of gonadal production, was higher in female than in male gonads of *P. lividus*, but in both sexes no differences were observed after exposure for 19 days to 8.0, 7.7 and 7.4 pH (Catarino et al. 2012). Conversely, in *E. mathaei* maintained for 6 weeks at similar pH values, a reduced spawning ability was observed in males kept at low pH, whereas in females both spawning ability and oocyte size did not change (Uthicke et al. 2013). In *Echinometra* sp. A, gonad index was not affected by a 77-day exposure at low pH, but a synergistic effect of decreased pH and increased temperature was revealed in both sexes, and males appeared more sensitive to low pH (Uthicke et al. 2014). After 9-month exposure to elevated pCO_2_, *Hemicentrotus pulcherrimus* showed a 1-month delay in gonad maturation and spawning, even though the maximum number of eggs was not affected (Kurihara et al. 2013). Gonad growth was reduced after 45- and 56-day exposure at low pH in *S. droebachiensis* (Siikavuopio et al. 2007; Stumpp et al. 2012) and after 140 days in *S. fragilis* (Taylor et al. 2014). Although several studies reported negative effects of reduced pH on sea urchin gonad growth, GSI reduction under low pH observed in this study in *P. lividus* females could be indicative of more detrimental effects arising from greater energy request to maintain homeostasis, with a consequent reduction in energy to invest in reproduction.

Overall, this study highlighted the presence of differences between sexes under control conditions and male and female sea urchins often responded differently to OA. As shown in CCA results, at 8.0 pH, sexes are clearly separated along the second canonical component, differing in CAT activity in both the digestive tract and gonads (higher in females), coelomocyte parameters (lysozyme activity in both coelomocytes and haemolymph, and coelomocyte volume higher in females) and metabolism (higher in males). In both sexes, a clear spatial distribution was observed according to pH values along the first canonical component. With increasing seawater acidity, enhanced CAT activity and decreased metabolism were observed in females. Females appeared more sensitive to pH variations with a clear separation between controls and reduced pH treatments. In females, low pH, 7.4 in particular, led to enhanced antioxidant defence in the digestive tract (with increased CAT activity), modified behaviour (with a reduction of righting time) and reduced energy investment in reproduction (with a gonadosomatic index decrease). Since the pattern of separation was opposite in females and males, the latter showed reduced antioxidant activity in gonads, reduced coelomocyte volume and lysozyme activity and increased metabolism. At extremely low pH condition (7.4), both males and females exhibited the highest value of coelomocyte number.

## Conclusions

In this study, different responses to OA were observed in males and females of *P. lividus*. However, responses appeared to be mostly influenced by basal differences between genders. Males exhibited lower protective levels in antioxidant and immune defence, and when subject to reduced pH they appeared to tackle this deficiency by somehow reducing metabolic expenditure. Conversely, females having a better basal protection against stress differently modulated antioxidant and immune-related responses but reduced their reproductive potential. Sex-specific differences, likely reflecting adaptive mechanisms of gametes with different life span, very short in sperm and long in eggs, can be important drivers affecting responses to environmental stress. For this reason, further research is needed to shed more light on the strengths and weaknesses of male and female sea urchins under global change scenarios.

## Electronic supplementary material

ESM 1 (PDF 943 kb)
