# Supplementary material for: Do males and females respond differently to ocean acidification? An experimental study with the sea urchin Paracentrotus lividus
Source: Environ Sci Pollut Res Int. 2020 Jul 10;27(31):39516–30. doi: 10.1007/s11356-020-10040-7 (PMC7524842; doi:10.1007/s11356-020-10040-7)
Supplement: Supplementary file 1 — (PDF 943 kb) [file 11356_2020_10040_MOESM1_ESM.pdf]

SPi Global

JOURNAL ROLLOUT DETAILS

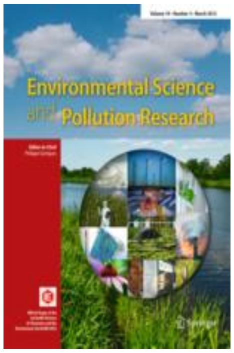

Environmental Science and Pollution Research

SPI CODE	**HES (11356)**
PROJECT OFFICER	**Dennis E. Villahermosa**
FREE COLOR THROUGHOUT (Y/N)	**Y**
LAYOUT (STANDARD/NON-STANDARD)	**Standard**

JOURNAL INSTRUCTIONS/UPDATES

AFFECTED PROCESS	INSTRUCTIONS
PREK/TYPESETTING/QA	Editorial articles: please capture the Author Biography and Photos
PREK/CED	If the provided “Highlights” is one sentence only per highlight, capture it as article note. Otherwise, query to PO.
PREK/CED/TYPESETTING	Insert Editorial Responsibility - Responsible Editor: Philipp Gariguess

REVISION HISTORY

Revision No.	Revision Date	Author	Revision Description
01		Ganabee Catalan	Initial Issue
02	04/26/10	Ganabee Catalan	Removal of entries which can be referred to TechInfo in the jobsheet
03	07/16/10	Ganabee Catalan	Removal of entries which can be referred to TechInfo in the jobsheet
04	06/16/12	Ganabee Catalan	New JRD format
05	05/16/13	Dennis Villahermosa	Re-phrase Editorial Responsibility
06	12/06/13	Dennis Villahermosa	Inclusion of Editorial Responsibilty in Spice
07	01/11/14	Dennis Villahermosa	Reviewed
8	02/08/14	Dennis Villahermosa	Instructions for Editorial articles
9	02/23/15	Dennis Villahermosa	Reviewed
10	01/15/18	Dennis Villahermosa	Reviewed
11	01/04/19	Dennis Villahermosa	Instruction for Political Neutrality Disclaimer SRG
12	06/03/19	Dennis Villahermosa	Instruction for Highlights section
